# Exploring the role of Hmox1 in ferroptosis and immune infiltration during renal ischemia-reperfusion injury

**DOI:** 10.1080/0886022X.2023.2257801

**Published:** 2023-09-19

**Authors:** Jin Luo, Jun Pei, Cheng-jun Yu, Xiao-mao Tian, Jie Zhang, Lian-ju Shen, Yi Hua, Guang-Hui Wei

**Affiliations:** a Department of Urology, Children’s Hospital of Chongqing Medical University, National Clinical Research Center for Child Health and Disorders, Ministry of Education Key Laboratory of Child Development and Disorders, Chongqing, China; b Chongqing Key Laboratory of Children Urogenital Development and Tissue Engineering, Chongqing, China

**Keywords:** Kidney transplantation, biomarkers, protein-protein interaction network, endogenous RNAs, immunological infiltration

## Abstract

Ischemia-reperfusion injury (IRI) is inevitable in kidney transplantations and, as a complex pathophysiological process, it can be greatly impacted by ferroptosis and immune inflammation. Our study aimed to identify the biomarkers of renal IRI (RIRI) and elucidate their relationship with immune infiltration. In this study, the GSE148420 database was used as a training set to analyze differential genes and overlap them with ferroptosis-related genes to identify hub genes using a protein–protein interaction (PPI) network, the least absolute shrinkage and selection operator (LASSO), and random forest algorithm (RFA). We verified the hub gene and ferroptosis-related phenotypes in a verification set and animal experiments involving unilateral IRI with contralateral nephrectomy in rats. Gene set enrichment analysis (GSEA) of single genes was conducted according to the hub gene to predict related endogenous RNAs (ceRNAs) and drugs to establish a network. Finally, we used the Cibersort to analyze immunological infiltration and conducted Spearman’s correlation analysis. We identified 5456 differential genes and obtained 26 ferroptosis-related differentially expressed genes. Through PPI, LASSO, and RFA, Hmox1 was identified as the only hub gene and its expression levels were verified using verification sets. In animal experiments, Hmox1 was verified as a key biomarker. GSEA of single genes revealed the seven most related pathways, and the ceRNAs network included 138 mRNAs and miRNAs. We predicted 11 related drugs and their three-dimensional structural maps. Thus, Hmox1 was identified as a key biomarker and regulator of ferroptosis in RIRI and its regulation of ferroptosis was closely related to immune infiltration.

## 1. Introduction

Ischemia-reperfusion injury (IRI) is a pathophysiological process that can exacerbate tissue damage when blood supply is restored after a period of tissue ischemia. This condition is commonly observed during major cardiovascular surgeries, cardiogenic shock, and organ transplantation [1,2]. The incidence of kidney failure and end-stage renal disease has increased in recent years, with kidney disease ranking among the top 10 fatal diseases in 2019. Kidney transplantation is in high demand as the ultimate treatment option for end-stage renal diseases [3]. Renal IRI (RIRI) is an inevitable consequence of kidney transplantation and can cause acute renal function loss and delayed recovery of chronic renal function [4]. Therefore, it is crucial to understand and manage IRI in order to improve the success rate of transplantation and minimize renal function loss.

Ferroptosis, a distinct type of programmed cell death, is characterized by iron overload and lipid peroxidation. It is regulated by various pathways, such as the inhibition of GPX4 and xCT [5], and has been implicated in various pathological processes, including acute kidney and liver injuries and tumor progression [6]. Regulation of ferroptosis is a promising target for managing IRI in kidney transplantation. Additionally, the immune response plays a crucial role in the development of RIRI. Zhao et al. found that inhibition of Toll-like receptor 4 could alleviate IRI and the associated graft function delay [7]. Loss of renal dendritic cells and activation of host adaptive immunity have long-term effects on IRI after allogeneic kidney transplantation [8]. However, the relationship between ferroptosis and immune cell infiltration in RIRI remains unclear.

This study aimed to identify the key genes involved in RIRI and their relationship with immune infiltration through bioinformatics analysis of differentially expressed ferroptosis-related genes. These findings provide novel insights into the intervention and regulation of the key genes involved in RIRI pathogenesis.

## 2. Methods

### 2.1. Dataset sources and processing

We conducted a comprehensive search of the Gene Expression Omnibus (GEO) database using the keywords ‘Renal’ and ‘IRI’ and chose mRNA expression data from four datasets to identify potential molecular markers linked to ferroptosis during RIRI. Dataset GSE148420 (GPL14746 platform) included four IRI and four sham groups, dataset GSE58438 (GPL11534 platform) included nine IRI and five sham groups, dataset GSE71647 (GPL7202 platform) included four IRI and four sham groups, and dataset GSE192532 (GPL21163 platform) included three IRI and three sham groups. We used an online bioinformatics tool (http://www.bioinformatics.com.cn) to transform homologous genes and ensure the homology of the results. The four datasets were normalized using the min-max approach, with GSE148420 serving as the training set and GSE58438, GSE71647, and GSE192532 as the external verification set. Using the Molecular Signature Database, we retrieved 38 genes related to ferroptosis.

### 2.2. Identification and analysis of DEFGs

We utilized the Deseq2 software package in R for computing the differentially expressed genes (DEGs) in the GSE1484 dataset. The significant indices were determined as the absolute value of Log2Fold > 1.0 and adjusted p-value < 0.05. To create heat maps and volcano plots of the DEGs, we used the online analytical toolkit Hiplot (https://hiplot.org), specifically designed for biomedical visualization [9]. The DEFGs were the result of the intersection between the DEGs and ferroptosis-related genes. Gene ontology (GO) and Kyoto gene encyclopedia and gene (KEGG) enrichment analyses served for exploring the potential biological roles and underlying mechanisms of the DEFGs under the assistance of the clusterProfiler R package. The results were mapped using the ggplot package in R. The GO enrichment analysis encompassed biological processes (BPs), cellular components (CCs), and molecular functions (MFs). KEGG provides insights into the advanced functions and utility regarding biological systems using molecular-level information. A p-value threshold of <0.05 was used to identify the major enrichment functions and pathways of DEFGs.

### 2.3. Hub gene discovery using PPI network, LASSO regression, and RFA in machine learning

We built a PPI network of DEFGs using the STRING database for exploring DEFG interactions more deeply. Cytoscape software served for visualizing the PPI network, with a confidence level of 0.4 as the cutoff value. The Degree Centrality (DC), Betweenness Centrality (BC), and Closeness Centrality (CC) evaluation indices of the CytoNCA plug-in were considered for identifying important genes in the PPI network of DEFGs. Furthermore, we utilized the LASSO regression algorithm in the glmnet R software package to precisely identify the target genes. We used the machine-learning algorithm RFA to perform regression and classification and generate models and screen the most important target genes using the R package. The key genes were then identified by intersecting the results from the PPI, LASSO, and RFA, defining them as the ferroptosis-related hub genes with the largest value in RIRIs. We utilized the Hiplot visual biomedical online analysis toolbox (https://hiplot.org) to draw a receiver operating characteristic (ROC) curve and verify its accuracy. An area under the curve (AUC) value greater than 0.7 was considered diagnostic [9,10]. Additionally, we analyzed the expression levels of the hub genes in the IRI and sham groups. Statistical significance was set at *p* < 0.05.

### 2.4. Establishment of the animal model

Forty adult male Sprague–Dawley rats (weight range: 220–250 g) were acquired from the Experimental Animal Center of Chongqing Medical University (SYXK [YU] 2022-0016, Chongqing, China). All rats were housed under identical conditions. Right nephrectomy was performed in all rats, who were classified into four groups (*n* = 10 for each group): sham, sham + Hmox1-inhibition, IRI, and IRI + Hmox1-inhibition. In the latter two groups, the left renal pedicle was directly split, and noninvasive vascular clipping was carried out for 40 min to induce ischemia. Successful ischemia resulted in a color change in the kidneys from brilliant red to purple black. The kidney color gradually returned to brilliant red after the vascular clip was removed, indicating effective reperfusion. In the sham group, the left renal pedicle was separated directly, and no ischemic treatment was administered. In the Hmox1 inhibition group, zinc protoporphyrin (ZnPP) (10 mg/kg, Sigma-Aldrich (#282820)) was injected intraperitoneally 2 h before surgery. After 24 h of reperfusion, all four groups of rats were anesthetized and sacrificed. The left kidneys were collected, and half of them were subjected to Western blotting (WB) to determine the expression of hub genes. The remaining kidneys were pathologically examined, and immunohistochemistry (IHC) was performed to assess kidney damage and gene expression in all groups. All animal experiments were approved by the Animal Ethics Committee of the Children’s Hospital Affiliated to Chongqing Medical University (IACUC Issue No. CHCMU-IACUC 20220429002).

### 2.5. Identification of histopathological injury in rats

Rats’ left kidneys were fixed in a paraformaldehyde solution and subjected to standard dehydration, transparency, embedding, and sectioning procedures. Hematoxylin and eosin (HE) staining was performed, and renal tubular injury was scored according to the Paller’s model [11]. More specifically, the renal cortex was observed at 400x magnification under a light microscope, and 10 areas were randomly selected in each high-power visual field. Ten renal tubules were randomly selected from each area, resulting in 100 renal tubular specimens per section. Renal tubular injury was assessed as follows: normal tubules received a score of 0; renal tubular conspiration with cell flattening received a score of 1; brush edge damage received a score of 1; shedding received a score of 2; epithelial cell necrosis received a score of 2; and tube formation received a score of 2. If the renal tubules displayed these pathological changes, the corresponding scores were recorded. If two or more pathological changes were present, the highest injury score was considered, with the total score for the 100 renal tubules considered as the final score. Serum creatinine (Scr) and Blood urine nitrogen (BUN) was measured using an automatic chemistry analyzer (BS-2000M, Mindray). Immunohistochemical staining was performed to evaluate renal tubular injury using kidney injury molecule-1 (KIM-1) as a marker. We conducted the EnVision two-step method as per the manufacturer’s instructions, with the antibody diluted 1:200. The average optical density (OD) method was used to score the samples, in which five non-overlapping visual fields were randomly selected for each specimen at 200x magnification. The ImageJ software served for image processing to measure the average OD. We treated the average OD value of five fields of view as the specimen’ OD value. Statistical analyses were performed using the Prism 9 software.

### 2.6. Verification of the hub gene

For confirming hub genes’ diagnostic value, we first normalized the gene expression data of GSE58438, GSE71647, and GSE58438 and synthesized them into a combined dataset using the min-max normalization method. ROC curves were then examined to determine accuracy, and hub genes with an AUC greater than 0.7 were considered diagnostic. *p* < 0.05 reported statistical significance. In addition, we used WB with the Hmox1 antibody from Proteintech (10701-1-AP) to determine hub gene expression in the renal IRI and sham groups. Kidney tissues from each group underwent 15 min of homogenization and centrifugation at 4 °C, 12,000 rpm, followed by collection of the supernatant. The bicinchoninic acid assay determined the protein concentration. Then, corresponding tissue proteins underwent sodium dodecyl sulfate-polyacrylamide gel electrophoresis, and were transferred onto a membrane, which was incubated in a primary antibody solution (1:1000 dilution) at 4 °C overnight. On the following day, the strips were washed with Tris-buffered saline containing 0.1% Tween-20 detergent, underwent 1 h of incubation of secondary antibody at room temperature (20 °C), and were washed again. The bands were assessed using chemiluminescence, and the figures were analyzed using ImageJ software. Statistical significance was set at *p* < 0.05 to determine the difference between groups using Prism 9.

### 2.7. Ferroptosis detection

IHC and WB were conducted to analyze the expression of ferroptosis-related proteins, specifically, ferritin heavy chain 1 (FTH1), acyl-CoA synthetase long-chain family member 4 (ACSL4), glutathione peroxidase 4 (GPX4), and cystine/glutamate antiporter (xCT). Malondialdehyde (MDA) and ferrous ions levels were also assessed. IHC staining was performed using the EnVision two-step method, with antibodies against GPX4 (381958), xCT (382036), ACSL4 (R24265), and FTH1 (381204) purchased from ZEN Bioscience and diluted to a 1:200 solution. Semi-quantitative analysis was carried out using ImageJ software, and the results were evaluated as previously described. For WB, the antibody was diluted to 1:1000; the further specific processes have been previously described. An MDA kit purchased from Elabscicence (E-BC-K025-M) served for measuring the relative MDA concentration (mol/g protein) as per the manufacturer’s instructions. Tissue lysates were prepared using a ferrous ion kit from Elabscience (E-BC-K773-M) to determine ferrous ion levels, and IHC staining was performed as previously described.

### 2.8. GSEA of single gene

We performed single-gene, gene set enrichment analysis (GSEA) using GSEA4.3.2, to identify differences between the IRI and sham groups in terms of the hub gene functions and pathways. The GSE148420 gene expression dataset was used in the present study. Additionally, we used the H and C2 sets from the Molecular Signatures Database to analyze 1000 genome sequences each time. The rats were divided into two groups based on their Hmox1 expression: high (IRI group) and low (sham group), and two phenotypes were established. We also used an absolute value of the normalized enrichment score greater than 1 and a significant gene enrichment threshold (*p* < 0.05) to identify significant gene sets.

### 2.9. Immune infiltration of the dataset and its correlation with the hub gene

We utilized the training set sample GSE148420 to assess the expressions of 22 immune cells in the IRI and sham groups using CIBERSORT (http://cibersort.stanford.edu/) [12]. The results were compared and visualized using the VioPlot R software package. We also performed Spearman’s correlation analysis between infiltrating immune cells and hub genes by virtue of Corrplot in R. Additionally, we measured the infiltration of 10 different immune cell types using an mMCP-counter [13]. A *p*-value < 0.05 was deemed statistically significant.

### 2.10. Construction of miRNA-hinge gene network and drug prediction map

We used ENCORI (https://starbase.sysu.edu.cn/index.php), a public RNA-RNA interaction website that analyzes thousands of CLIP-seq high-throughput sequencing data, to obtain competing endogenous RNA (ceRNA) data. Cytoscape served for the construction of the network map [14]. The hub gene was associated with ceRNA through miRNA, and the relationship between miRNA and ceRNA was visualized by drawing a Sankey diagram using the ggalluvial R package. Additionally, we used PubChem (https://pubchem.ncbi.nlm.nih.gov/), the National Institutes of Health public database of chemical drugs, for predicting the structure of drugs and molecular compounds that could interact with hub genes.

## 3. Results

### 3.1. Identification and enrichment of DEFGs

**Figure 1.**
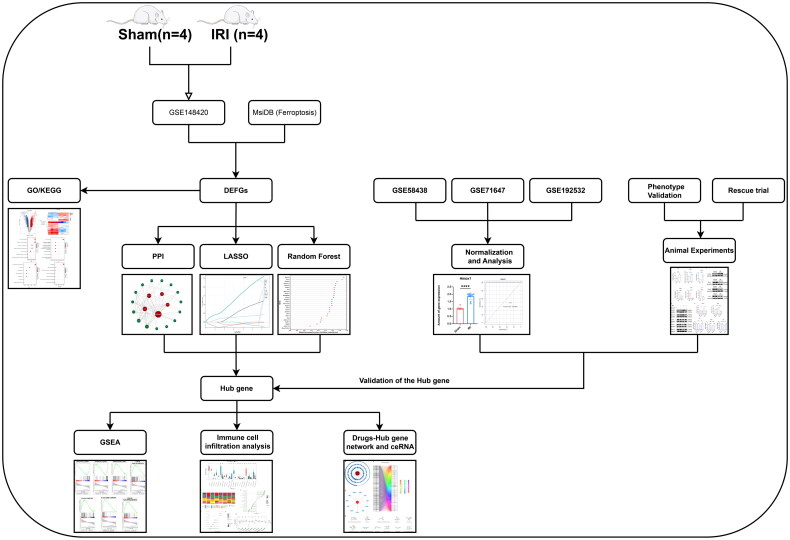
Research workflow.

**Figure 2.**
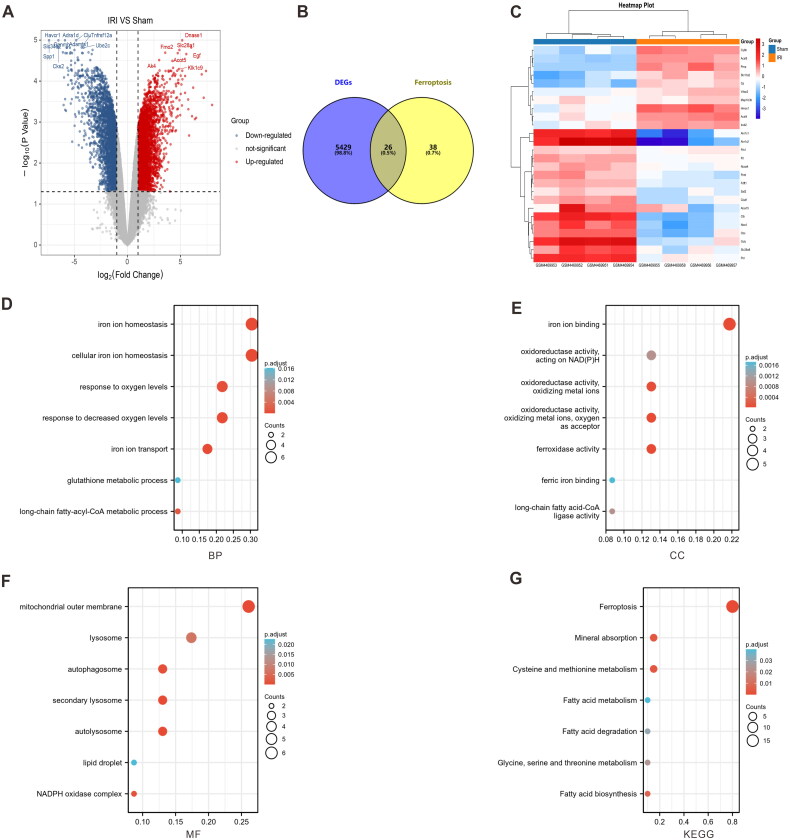
Identification and analysis of the differentially expressed ferroptosis-related genes (DEFGs). (A) The differentially expressed genes (DEGs) volcano map of the GSE148420 dataset; (B) the gene heatmap of DEFGs; (C) Venn plot of DEFGs; (D) the biological process (BP) of GO enrichment analysis plot of DEFGs; (E) the cellular component (CC) of GO enrichment analysis plot of DEFGs; (F) the molecular function (MF) of GO enrichment analysis plot of DEFGs; and (G) the Kyoto encyclopedia of genes and genomes (KEGG) enrichment analysis plot of DEFGs.

Figure 1 shows the flowchart of the investigation. The training set GSE 148420 included 5456 DEGs (Log2Fold absolute value > 1.0; adjusted *p* < 0.05), 2486 upregulated GSEs, and 2970 downregulated GSEs in the IRI group. The distribution of these DEGs is presented using a volcano plot (Figure 2A). To determine the DEFGs, we obtained 38 ferroptosis-related genes from the Molecular Signatures Database and intersected them with DEGs related to IRI, resulting in 26 DEFGs (Figure 2B). The heatmap illustrates that the DEFGs could effectively distinguish IRI from the sham group (Figure 2C). The GO enrichment analysis of DEFGs revealed significant enrichment in biological processes related to the metabolism of iron ions and glutathione as well as hypoxia. Cellular components were primarily associated with the outer mitochondrial membrane, autophagy, and lipid transport. The primary molecular function of DEFGs was identified as iron–ion activity. Furthermore, KEGG pathway analysis indicated the primary enrichment of the DEFGs in ferroptosis and fatty acid metabolism (Figure 2D-G).

### 3.2. Identification of the hub gene based on PPI, LASSO regression, and RFA

To identify the proteins that interact with the 26 identified DEFGs, we entered their information into the STRING database. The results were imported into Cytoscape to create a PPI network (Figure 3A, B). We identified the top five important genes by combining the CytoNCA plugin with the DC, BC, and CC evaluation indices. The most frequently identified genes were Hmox1, Ireb2, Slc11a2, Cosd1, and Fth1. LASSO regression analysis identified the following six key genes: Fdft1, Ftl, Por, Acsl5, Hmox1, and Acsl4 (Figure 3C, D). We identified the top five of them as important genes. Finally, we used a RFA and found that the top five most important genes were Nox4, Por, Acsl5, Hmox1, and Vdac2 (Figure 3E). Thus, we concluded that Hmox1 was deemed a hub gene based on the intersection Venn diagram of the genes obtained using the above three methods (Figure 3F).

Figure 3.Identification of hub genes based on PPI, LASSO, and random forest algorithm. (A, B) Protein–protein interaction (PPI) network analysis (DC, BC, and CC; red indicates the key genes for screening); (C, D) the least absolute shrinkage and selection operator (LASSO) regression analysis in the GSE148420 dataset; (E) the top genes determined using the random forest algorithm; (F) the Venn plot of key gene intersection determined using the PPI, LASSO, and random forest algorithms; (G) the receiver operator characteristic (ROC) curve analysis plot of the Hub genes; and (H) the statistical plot of the expression of the hub genes between the ischemia-reperfusion injury (IRI) and sham groups. (**** represents *p*-value < 0.0001).

**Figure:**
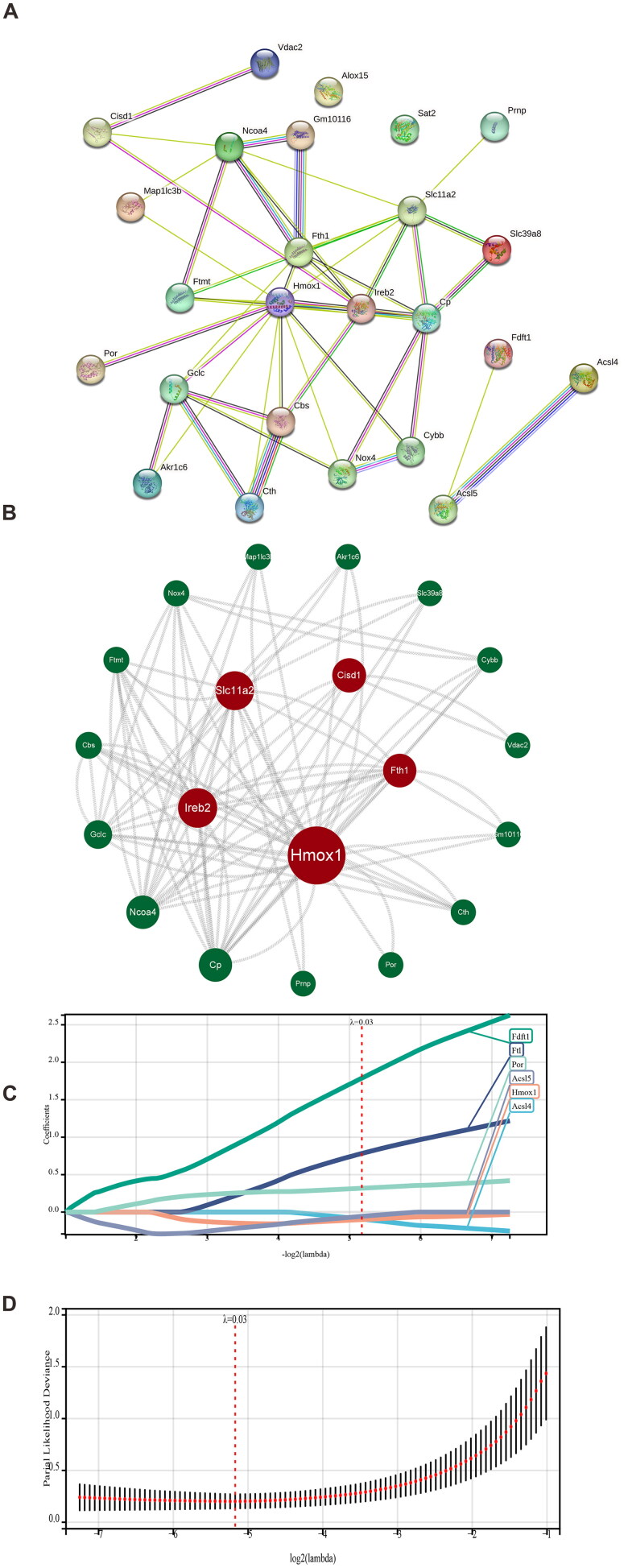

**Figure:**
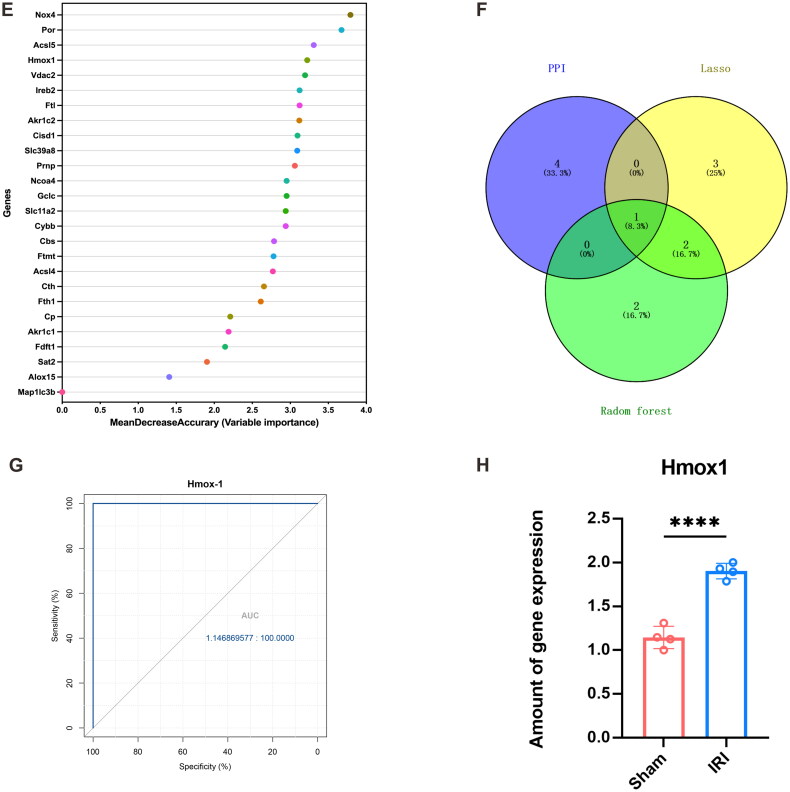

We measured the sensitivity and specificity exhibited by the diagnostic prediction model using the AUC, which confirmed the reliability of Hmox1 as a hub gene. The hub gene AUC in the training set GSE148420 was higher than 0.7 (Figure 3G). According to the results, the p-value for the statistical comparison of gene expression between the IRI and sham groups was less than 0.05 (Figure 3H). Therefore, we concluded that Hmox1 critically impacted the pathophysiology of renal IRI.

### 3.3. Assessment of kidney histopathological damage

**Figure 4.**
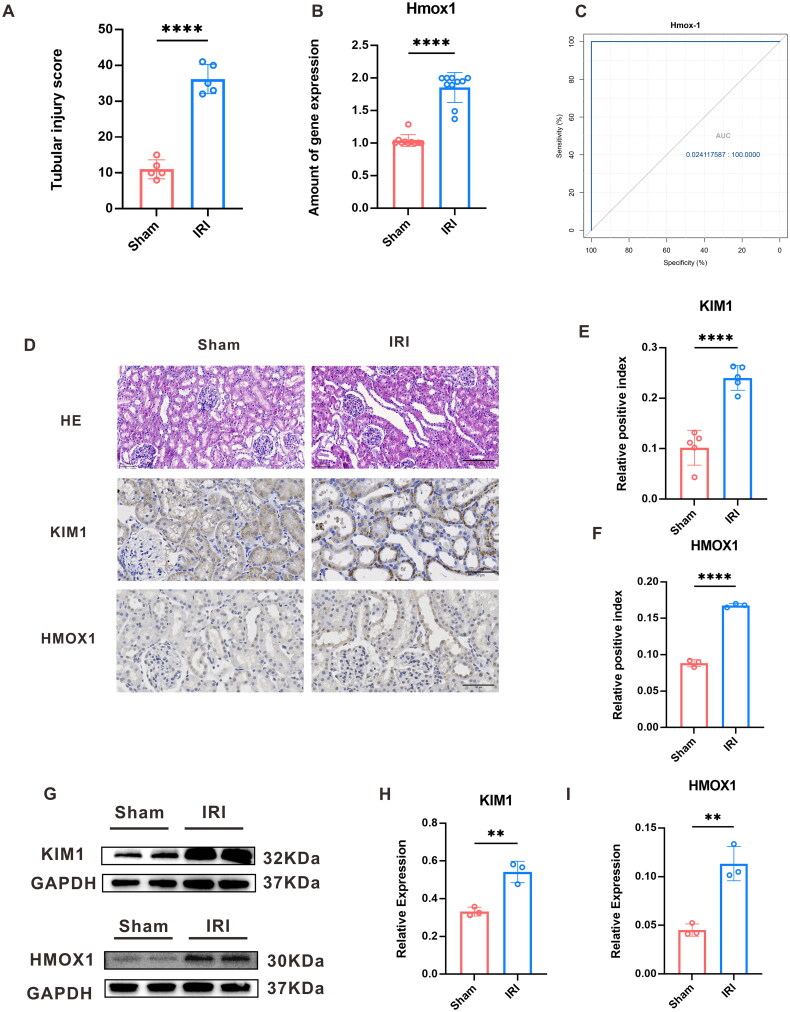
Validation of the hub genes and animal models. In the validation sets GSE58438, GSE71647, and GSE192532; (A) the tubular injury score chart; (B) the expression statistics of *Hmox1* in the Sham and IRI groups; (C) the ROC curve analysis plot of *Hmox1*; (D) immunohistochemistry (IHC) of the *Hmox1* and the renal tubular injury marker KIM-1 in rat renal tissue, as well as hematoxylin-eosin staining; (E, F) average optical density statistical plot of KIM-1 and *Hmox1* IHC positive expression, respectively; (G) Western Blot protein band plot of KIM-1 and *Hmox1* in renal tissues under the RIRI model; and (H, I) and the proteins’ statistical expression in the sham and IRI groups, respectively (**** represents *p*-value < 0.0001, and ** represents *p*-value < 0.01).

To evaluate the pathological injury resulting from RIRI, we used HE staining and IHC. HE staining revealed normal glomerular and tubular structures in the sham group under a light microscope. In contrast, the renal tubules were disorganized and noticeably dilated in the IRI group. Renal tubule epithelial cells were exfoliated, and there were necrosis and fragments in the lumen. The renal tubular damage score was considerably higher in the IRI group than that in the sham group (Figure 4A, D). Moreover, the IRI group presented a higher expression of KIM-1, a hallmark of renal tubular damage relative to the sham group (Figure 4D, E, G).

### 3.4. Verification of hub gene

After conducting the aforementioned analysis, we identified Hmox1 as a potential biomarker associated with ferroptosis during RIRI. We evaluated the accuracy of the hub gene expression in the external validation sets GSE58438, GSE71647, and GSE192532, by examining the ROC curve. The AUC was found to be higher than 0.7, confirming its accuracy (Figure 4C). Additionally, we performed statistical analysis and standardization of hub gene expression levels in the IRI and sham groups. We discovered that the hub gene expression trends were comparable between the validation and training sets, and their expression presented an obvious difference in statistics between the groups (*p* < 0.05) (Figure 4B).

Moreover, we used WB and IHC staining in a rat RIRI model to confirm our findings. We found that animals in the IRI group presented a higher Hmox1 expression relative to the sham group (Figure 4D, F, G, 1).

### 3.5. Verification of ferroptosis markers and rescue experiments

**Figure 5.**
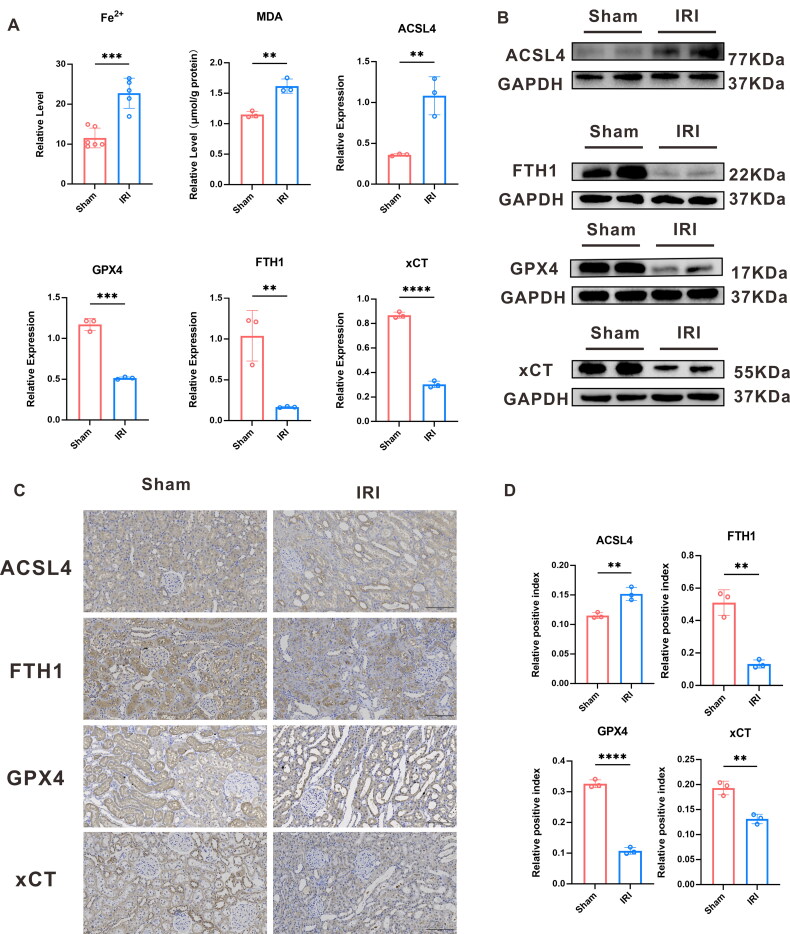
Verification of ferroptosis markers. (A) The relative level of ferrous ions, MDA, and the ACSL4, FTH1, GPX4, and xCT proteins expressed statistically in the sham and IRI groups; (B), (C), and (D) ACSL4, FTH1, GPX4, and xCT immunohistochemical staining findings, along with a statistical analysis of the relative expression of these proteins in the renal tissues using the RIRI model.

Ferroptosis is a complex process that involves numerous molecules related to iron overload and lipid peroxidation. Among them, GPX4, FTH1, and xCT are commonly used to inhibit ferroptosis, and an increase in ACSL4 expression indicates ferroptosis. WB analysis of these markers revealed significant differences in their expression between the IRI and sham groups, with decreased levels of GPX4, FTH1, and xCT and increased levels of ACSL4 in the former (Figure 5A, B), which conformed to the results of the IHC analysis (Figure 5C, D). Furthermore, we observed remarkably increased MDA levels in the IRI group using the MDA kit, and an elevated level of ferrous ions using the ferrous ion detection kit (Figure 5A).

**Figure 6.**
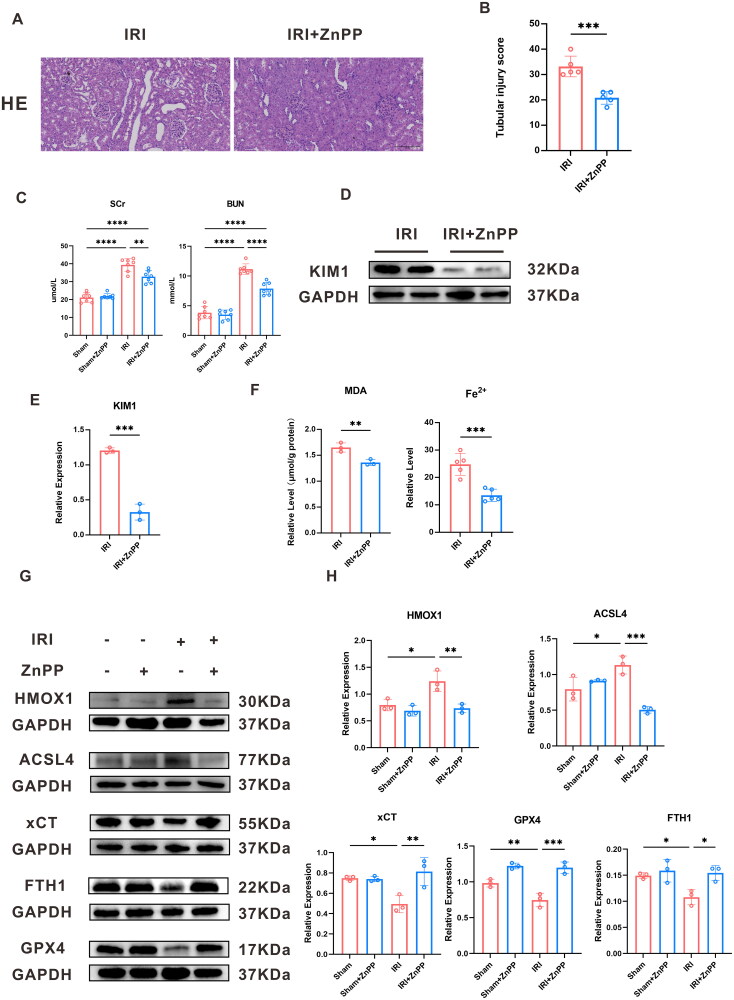
Rescue experiments. (A), (B) HE staining and tubular injury score of IRI rescue experiments. (C)The relative level of Scr and BUN in four group. (D), (E) Western Blot protein band plot of KIM-1 and the protein’s statistical expression in the IRI and IRI + ZnPP groups. (F) The relative level of ferrous ions, MDA. (G) The western blot protein band plot of *Hmox1*, ACSL4, FTH1, GPX4, and xCT in renal tissues in the rescue experiments. (H) Statistical analysis of the western blot proteins from *Hmox1*, ACSL4, FTH1, GPX4, and xCT. (*** represents *p*-value < 0.001, ** represents *p*-value < 0.01, and * represents *p*-value < 0.05.)

Additionally, we inhibited Hmox1 expression using ZnPP to examine the interplay between Hmox1 and ferroptosis and the protective effect of RIRI. The relative level of MDA, ferrous ions, Scr, BUN, and Tubular injury score decreased (Figure 6A, B, C, F). WB analysis indicated that the ACSL4 and KIM1 expression decreased, while expressions of GPX4, FTH1, and xCT increased in the IRI + Hmox1-inhibition group (Figure 6D, E, F, G) (p-value < 0.05).

### 3.6. Single gene GSEA analysis of the hub gene

**Figure 7.**
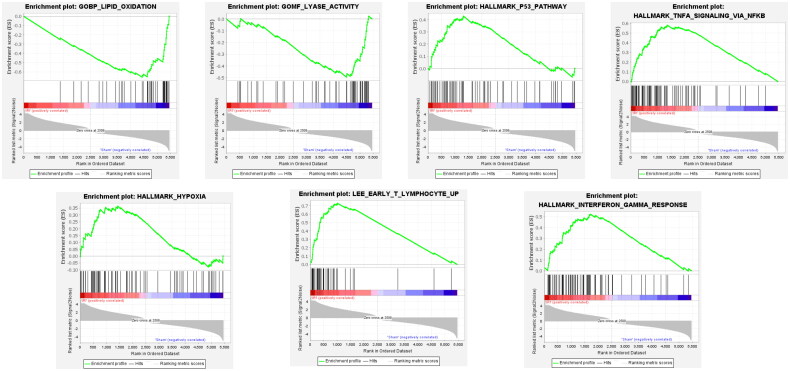
The signaling pathway associated with the *Hmox1* gene based on GSEA.

GSEA analysis revealed that the Hmox1 expression was negatively related to lipid oxidation and lyase activity, whereas early T-lymphocytes, gamma interferon response, tumor protein p53, tumor necrosis factor-α stimulated nuclear factor kappa B (NF-κB) pathway, and hypoxia were positively correlated with Hmox1 expression (Figure 7). Previous studies have shown that hypoxia, lipid oxidation, tumor protein p53, and NF-κB signaling pathways were all key pathways involved in inducing ferroptosis in tissues, while early T-lymphocyte and interferon gamma responses were closely associated with T-cell activation.

### 3.7. Analysis of correlation between hub gene and immune infiltration

**Figure 8.**
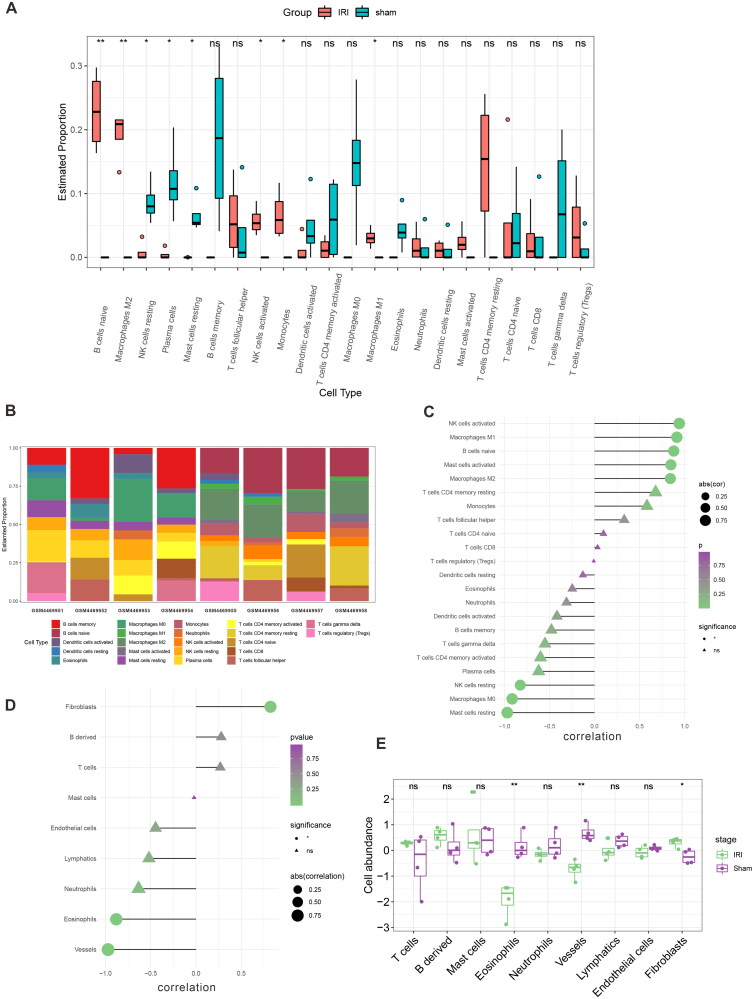
Immune cell infiltration and correlation analysis of the hub gene with immune cells. Based on CIBERSORT, (A) the expression of 22 different immune cell types in the training set GSE148420 of the IRI and sham group; (B) a stacked plot of the expression of 22 different immune cell types in each sample; (C) and a correlation analysis plot between *Hmox1* and 22 different immune cell types. Based on mMCP, (D) a correlation analysis plot between nine different immune cell types and (E) the expression of nine different immune cell types in the training set.

GSEA of the hub genes was carried out to investigate their role in RIRI, which revealed that immune cells were involved in this process. To confirm this finding, we conducted a CIBERSORT immune infiltration analysis and found significant differences in the abundance of the eight types of immune cells between the IRI and sham groups (p-value < 0.05). These cells included naive B-cells, resting natural killer (NK) cells, M1 and M2 macrophages, resting mast cells, activated NK cells, plasma cells, and monocytes (Figure 8A, B). Using mMCP analysis, we also observed differences in the abundance of fibroblasts, eosinophils, and vessels (Figure 8E).

Spearman’s correlation analysis was utilized to obtain a more precise evaluation of the relationship between hub genes and immune cell infiltration, confirming the positive relevance of Hmox1 to the infiltration of immune cells, such as active NK cells, naive B-cells, M1 and M2 macrophages, and resting mast cells, and its negative relevance to NK cells, plasma cells, and monocytes (Figure 8C). In the mMCP group, we found that fibroblasts were positively correlated, whereas eosinophils and vessels were negatively correlated (Figure 8D).

### 3.8. Construction of ceRNA network of hub gene and related drugs prediction

After analyzing 138 ceRNAs related to the hub genes using the ENCORI database, we found that all of them encoded proteins and constructed a network based on these miRNAs (Figure 9A, D). Additionally, using the PubChem database, we identified 11 potential drugs or molecules that may interact with the hub genes (Figure 9B). The three-dimensional structures of these compounds are displayed in Figure 9C, which could assist in examining the hub genes more deeply.

Figure 9.Prediction of the ceRNA network of hub genes and related drugs. The ceRNA map of *Hmox1* is shown in (A), and the hub gene is highlighted in red, while associated mRNAs are shown in blue; (B) action network diagram of pharmaceuticals connected to the *Hmox1* gene; (C) and 3D structure diagram of *Hmox1* gene-related drugs. Sankey diagram of the ceRNA network (D), with mRNAs on the left and corresponding miRNAs on the right.

**Figure:**
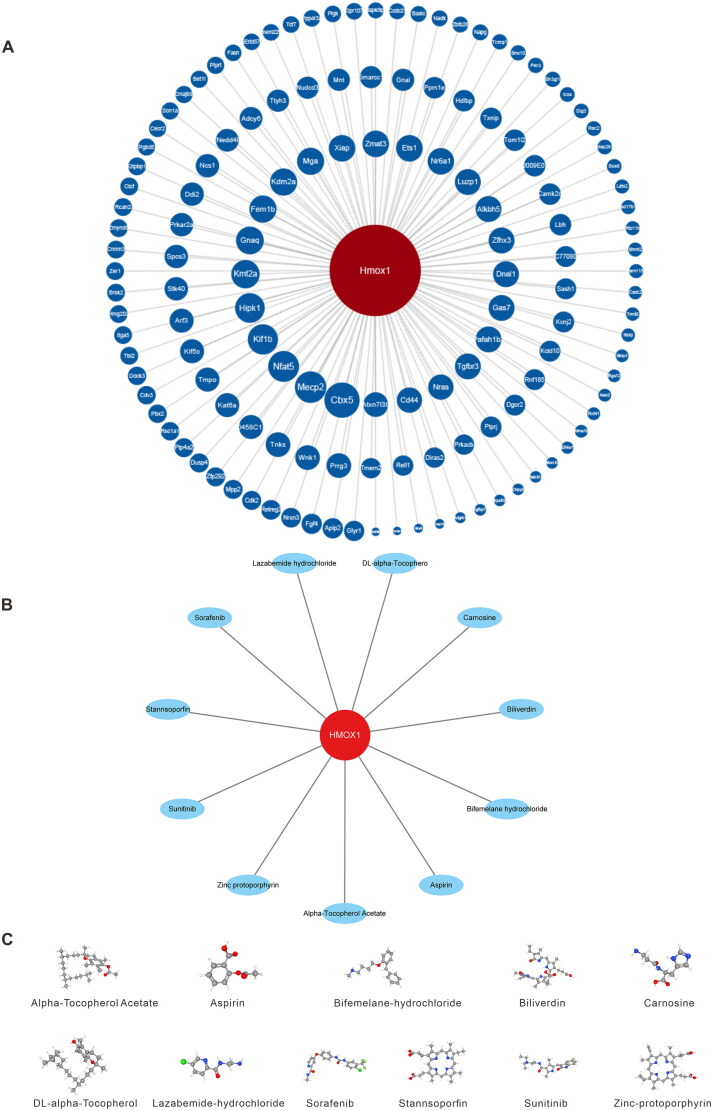

**Figure:**
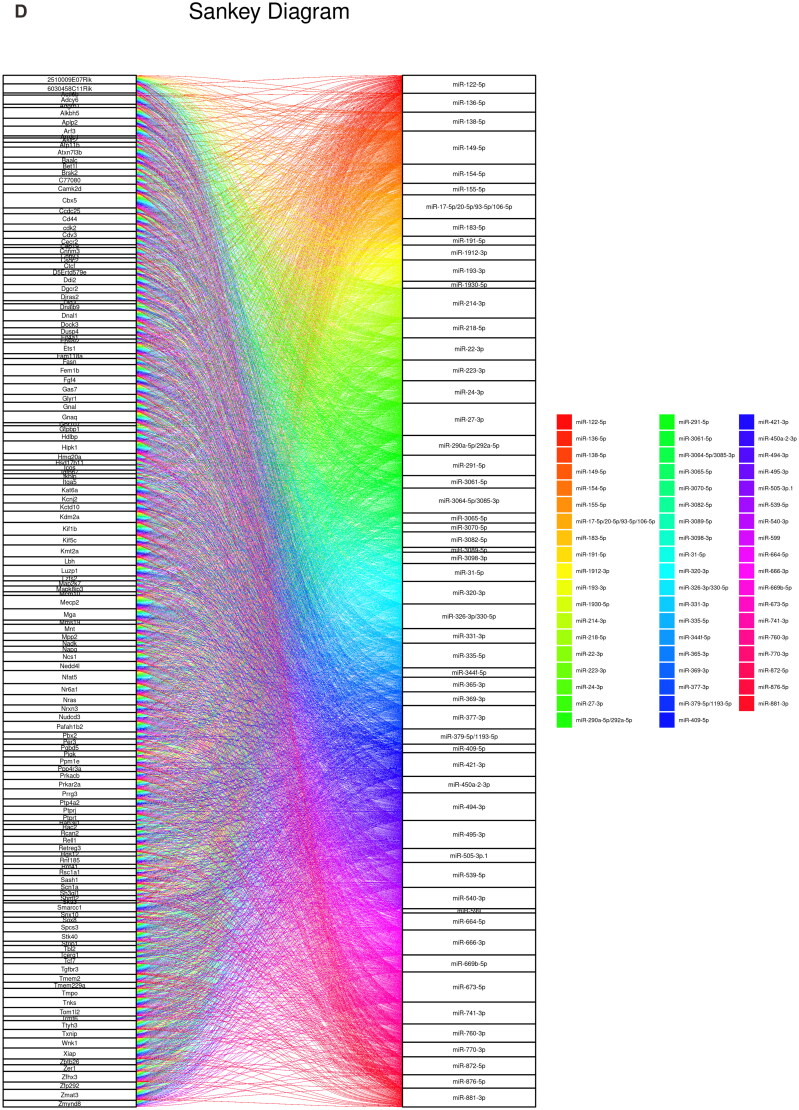

## 4. Discussion

RIRI is a significant cause of acute kidney injury, capable of significantly impairing kidney function and increasing mortality by 15% in severe cases [15]. Early diagnosis, proper classification, and prompt treatment are vital to reduce these effects. Despite the complex RIRI pathogenesis, bioinformatical analysis can assist in understanding the molecular mechanisms associated with RIRI initiation and help the development and exploration of potential therapeutic targets. Since 2012, ferroptosis, a type of PCD, has been shown to play a critical role in various acute kidney injuries [5,16]. Friedmann et al. demonstrated that Gpx4 knockout can induce acute renal failure in mice, highlighting that characteristics related to ferroptosis can be reversed by ferroptosis inhibitors [17]. Further, Su et al. considered the relationship between ferroptosis and kidney injury in IRI *via* mitochondrial oxidation and autophagy [18]. However, the key genes and molecular markers associated with ferroptosis in RIRI have not yet been identified. In addition, immune system activation crucially impacts the RIRI, and identifying appropriate diagnostic markers and immune cell infiltration patterns helps alleviate and prevent RIRI [19]. In this study, we aimed to use bioinformatics to screen for molecular markers of ferroptosis during RIRI. Cibersort and mMCP analyses serve for analyzing immune cell infiltration patterns in diseases. We are committed to identifying diagnostic markers of ferroptosis-related genes in RIRI and exploring the effect of immune cell infiltration.

To investigate the molecular markers related to ferroptosis during RIRI, we downloaded gene expression data from the GEO database and divided them into training and verification sets. Differential gene expression analysis revealed 5456 DEGs under a given threshold. To further identify genes related to ferroptosis, we obtained 38 genes from molecular characteristic databases and found 26 DEGs that intersected with these genes, which we defined as DEFGs. GO and KEGG enrichment analysis revealed the primary enrichment of the 26 DEFGs in hypoxia, lipid metabolism, and iron ion metabolism, suggesting that hypoxia-related factors and ferroptosis play critical roles in RIRI.

To identify key genes that play a pivotal role in IRI and ferroptosis, we employed three different screening methods: PPI, LASSO regression, and RFA. PPI is a classic approach for understanding and providing insights into protein interactions, while LASSO regression is a machine learning algorithm that selects variables from sample data using a penalty approach and compresses the original coefficients to zero, deeming variables with these coefficients as non-significant [20]. Finally, RFA is an ensemble-learning algorithm that uses the voting or averaging of the results from several weak classifiers to produce outcomes with good accuracy and generalization performance for the entire model [21].

By employing all three methods, our analysis identified Hmox1, a gene that encodes heme oxygenase 1, as the most valuable pivotal gene in RIRI. Validation sets GSE58438, GSE71647, and GSE192532 showed a significant increase in Hmox1 expression, consistent with the training set, indicating its reliability. Upon further investigation in a rat model, we observed clear renal tubular damage in the RIRI group, as confirmed by HE staining and IHC using KIM-1. Additionally, WB analysis of kidney tissues revealed significantly higher levels of Hmox1 expression in the IRI group relative to the sham group. These findings conform to previous studies, revealing that IRI significantly exacerbates histopathological damage to the kidneys. Hence, the expression of Hmox1 is positively correlated with the occurrence of kidney injury. Furthermore, we observed significant differences in biomarkers related to ferroptosis, such as ACSL4 and GPX4, and an increase in MDA and ferrous ion levels, which suggest iron overload and lipid peroxidation, and indicate that ferroptosis occurred during RIRI.

According to previous studies, Hmox1 exerts a critical effect in ferroptosis by protecting the body from hypoxia and infection of myocardial osteosarcoma cells, breast epithelium in mastitis, and cardiomyocytes in sickle cell disease [22–25]. Furthermore, Hmox1 has been demonstrated as a protective factor in AKI [26]. Thus, we carried out animal experiments using ZnPP, a specific Hmox1 inhibitor, to determine the regulatory relationship between Hmox1 and ferroptosis. The results showed significant differences in the ferroptosis-related proteins produced in the sham and IRI groups, and these changes were rescued by the injection of ZnPP. This suggests that Hmox1 may regulate ferroptosis in RIRI and may be a potential biomarker for RIRI.

Our study aimed at exploring the biological role of Hmox1 in RIRI. According to GSEA, Hmox1 expression exhibited a positive relevance to hypoxia, lipid oxidation, P53, and NF-κB, which are closely related to ferroptosis, which underscores the crucial role of Hmox1 in ferroptosis [27, 28]. In addition, correlation analysis revealed that Hmox1 was positively correlated with NK cells, naive B-cells, and M1 and M2 macrophages, suggesting that it induces the expression of immune-related molecules and activates the local immune system. Furthermore, the correlation between fibroblasts and vessels suggests that fibrosis is caused by IRI, which may involve immune cells induced by Hmox1. During acute RIRI, immune cells are activated and recruited into the injured kidney tissue. This process is influenced by several variables, including enhanced adhesion molecule expression, chemokine and cytokine secretion, complement system activation, and elevated renal vascular endothelial permeability. Neutrophils, dendritic cells, macrophages, and lymphocytes from the congenital and adaptive immune systems are thought to be involved in these lesions, further demonstrating that RIRI is an inflammatory illness mediated by the immune system [29]. Macrophages vitally impact the immune response by providing both innate and cellular immunity. More specifically, the tissue microenvironment confirms macrophage differentiation, transcriptome, and function, and thus, immune function is accomplished by polarizing M1 and M2 in different ways [29].

The kidney microenvironment is regionally heterogeneous; it is strongly dynamic and decided by local pathological and physiological conditions. According to a 2017 study, a high-salt microenvironment in the renal medulla could induce mononuclear phagocyte recruitment and enhance antibacterial function, which may assist in preventing urinary tract infections [30]. During renal injury, renal macrophages play an important but dichotomous role in the damage response. Proinflammatory macrophages are predominant in the early injury stage and benefit the initial kidney damage, while activated macrophages dominate in the later stages and benefit the tubular cell regeneration [31]. Granulocyte-macrophage colony-stimulating factor secreted by renal tubular cells can also induce the phenotypic conversion of macrophages [32]. Recent studies have shown that drugs targeting S100a8/a9hi macrophage subsets can alleviate acute RIRI [33]. Our analysis of immune infiltration suggests that Hmox1 expression is positively correlated with both M1 and M2 macrophages, indicating its involvement in macrophage-mediated mechanisms of RIRI. However, the underlying mechanisms require further investigation.

Furthermore, research on ceRNAs and miRNAs is currently a hot topic, and the interactions between ceRNAs and hub genes have attracted the attention of researchers. Our study focused on building a ceRNA network and screened ceRNAs related to Hmox1 and their shared miRNAs. These ceRNAs may participate in the regulation of Hmox1 through competitive binding of miRNA or other pathways or respond to changes in Hmox1 and produce biological effects. Lee et al. reported that FASN and Hmox1 are involved in regulating nonalcoholic fatty liver disease, and miRNA-539 can inhibit IRI-induced mitochondrial fission in myocardial cells [34,35]. However, the specific molecular underpinnings of the interactions between ceRNAs, miRNAs, and RIRI have not yet been fully elucidated. As such, it is necessary to conduct further studies to map out the influence of these interactions on RIRI.

Finally, our study confirmed the involvement of 13 medicines or chemical compounds, some of which may be prospective anti-RIRI medications, in hub gene regulation. The effects of medications and chemical substances on IRI have been studied extensively. For instance, aspirin can indirectly affect myocardial IRI through its antiplatelet function, and alpha tocopherol has a protective effect against spinal cord IRI and inhibits ferroptosis in the liver IRI [36,37]. Although there have been no comparable studies on renal IRIs, these medications or chemical compounds have the potential to be prescribed for the RIRI treatment.

## 5. Conclusion

In this study, we used PPI, LASSO regression analysis, and the random forest procedure to screen for ferroptosis-related biomarkers in RIRI. We also conducted a comprehensive analysis of *Hmox1* expression, ferroptosis, and immune infiltration in RIRI using CIBERSORT and mMCP. Furthermore, we used ZnPP to inhibit HMOX1, examined its regulation of ferroptosis, and explored the related information on ceRNAs and drugs. However, this study has some limitations. First, our analysis relied on the secondary mining of previously published datasets. Different datasets and analytical approaches may lead to different conclusions. Additionally, the small sample size used in this bioinformatics study may have resulted in less accurate results. It is important to note that omissions or errors may have occurred during the analysis of homologous gene transformations using mouse genes. Thus, it is necessary to conduct experiments with larger sample sizes to confirm our findings. In addition, this study lacked relevant data from human clinical studies, which will be the focus of our future research on RIRI.

## References

[1] Nieuwenhuijs-Moeke GJ, Pischke SE, Berger SP, et al. Ischemia and reperfusion injury in kidney transplantation: relevant mechanisms in injury and repair. J Clin Med. 2020;9(1):1. doi:10.3390/jcm9010253. PMC7019324 31963521

[2] Gueler F, Gwinner W, Schwarz A, et al. Long-term effects of acute ischemia and reperfusion injury, in. Kidney Int. 2004;66(2):523–17. doi:10.1111/j.1523-1755.2004.761_11.x. 15253702

[3] World Health Organisation. Disease Burden and Mortality Estimates; 2019 [cited 2023 April 3]. https://www.who.int/data/gho/data/themes/mortality-and-global-health-estimates.

[4] Ponticelli C. Ischaemia-reperfusion injury: a major protagonist in kidney transplantation. Nephrol Dial Transplant. 2014;29(6):1134–1140. doi:10.1093/ndt/gft488. 24335382

[5] Dixon SJ, Lemberg KM, Lamprecht MR, et al. Ferroptosis: an iron-dependent form of nonapoptotic cell death. Cell. 2012;149(5):1060–1072. doi:10.1016/j.cell.2012.03.042. 22632970 PMC3367386

[6] Su L, Jiang X, Yang C, et al. Pannexin 1 mediates ferroptosis that contributes to renal ischemia/reperfusion injury. J Biol Chem. 2019;294(50):19395–19404. doi:10.1074/jbc.RA119.010949. 31694915 PMC6916502

[7] Zhao H, Perez JS, Lu K, et al. Role of toll-like receptor-4 in renal graft ischemia-reperfusion injury. Am J Physiol Renal Physiol. 2014;306(8):F801–F811. doi:10.1152/ajprenal.00469.2013. 24523386 PMC3989634

[8] Ozaki KS, Kimura S, Nalesnik MA, et al. The loss of renal dendritic cells and activation of host adaptive immunity are long-term effects of ischemia/reperfusion injury following syngeneic kidney transplantation. Kidney Int. 2012;81(10):1015–1025. doi:10.1038/ki.2011.458. 22278023 PMC3340432

[9] Li J, Miao B, Wang S, et al. Consortium, Hiplot: a comprehensive and easy-to-use web service for boosting publication-ready biomedical data visualization. Brief Bioinform. 2022;23(4):bbac261–288. doi:10.1093/bib/bbac261. 35788820

[10] Wu J, Zhang H, Li L, et al. A nomogram for predicting overall survival in patients with low-grade endometrial stromal sarcoma: a population-based analysis. Cancer Commun. 2020;40(7):301–312. doi:10.1002/cac2.12067. PMC7365459 32558385

[11] Paller MS, Neumann TV, Knobloch E, et al. Reactive oxygen species and rat renal epithelial cells during hypoxia and reoxygenation. Kidney Int. 1991;40(6):1041–1049. doi:10.1038/ki.1991.312. 1662318

[12] Newman AM, Steen CB, Liu CL, et al. Determining cell type abundance and expression from bulk tissues with digital cytometry. Nat Biotechnol. 2019;37(7):773–782. doi:10.1038/s41587-019-0114-2. 31061481 PMC6610714

[13] Petitprez F, Levy S, Sun C-M, et al. The murine microenvironment cell population counter method to estimate abundance of tissue-infiltrating immune and stromal cell populations in murine samples using gene expression. Genome Med. 2020;12(1):86. doi:10.1186/s13073-020-00783-w. 33023656 PMC7541325

[14] Li JH, Liu S, Zhou H, et al. StarBase v2.0: decoding miRNA-ceRNA, miRNA-ncRNA and protein-RNA interaction networks from large-scale CLIP-Seq data. Nucleic Acids Res. 2014;42:D92–D97. doi:10.1093/nar/gkt1248. PMC3964941 24297251

[15] Bonventre JV, Weinberg JM. Recent advances in the pathophysiology of ischemic acute renal failure. J Am Soc Nephrol. 2003;14(8):2199–2210. doi:10.1097/01.ASN.0000079785.13922.F6. 12874476

[16] Li J, Cao F, Yin H L, et al. Ferroptosis: past, present and future. Cell Death Dis. 2020;11(2):88–100. doi:10.1038/s41419-020-2298-2. 32015325 PMC6997353

[17] Friedmann Angeli JP, Schneider M, Proneth B, et al. Inactivation of the ferroptosis regulator Gpx4 triggers acute renal failure in mice. Nat Cell Biol. 2014;16(12):1180–1191. doi:10.1038/ncb3064. 25402683 PMC4894846

[18] Su L, Zhang J, Gomez H, et al. Mitochondria ROS and mitophagy in acute kidney injury ABSTRACT. Autophagy. 2023;19(2):401–414. doi:10.1080/15548627.2022.2084862. 35678504 PMC9851232

[19] Jang HR, Rabb H. Immune cells in experimental acute kidney injury. Nat Rev Nephrol. 2015;11(2):88–101. doi:10.1038/nrneph.2014.180. 25331787

[20] Tibshirani R. Regression shrinkage and selection via the lasso: a retrospective. J R Stat Soc Series B Stat Methodol. 2011;73:273–282. doi:10.1111/j.1467-9868.2011.00771.x.

[21] Breiman L. Random forests. Mach Learn. 2001;45(1):5–32. doi:10.1023/A:1010933404324.

[22] Lin H, Chen X, Zhang C, et al. EF24 induces ferroptosis in osteosarcoma cells through HMOX1. Biomed Pharmacother. 2021;136:111202. doi:10.1016/j.biopha.2020.111202. 33453607

[23] Zhang Q, Bai X, Lin T, et al. HMOX1 promotes ferroptosis in mammary epithelial cells via FTH1 and is involved in the development of clinical mastitis in dairy cows. Antioxidants. 2022;11(11):2221. doi:10.3390/antiox11112221. 36421410 PMC9686786

[24] Menon AV, Liu J, Tsai HP, et al. Excess heme upregulates heme oxygenase 1 and promotes cardiac ferroptosis in mice with sickle cell disease. Blood. 2022;139(6):936–941. doi:10.1182/blood.2020008455. 34388243 PMC8832481

[25] Wilks A. Heme oxygenase: evolution, structure, and mechanism. Antioxid Redox Signal. 2002;4(4):603–614. doi:10.1089/15230860260220102. 12230872

[26] Li Y, Ma K, Han Z, et al. Immunomodulatory effects of heme oxygenase-1 in kidney disease. Front Med (Lausanne). 2021;8:708453. doi:10.3389/fmed.2021.708453. 34504854 PMC8421649

[27] Liu Y, Gu W. p53 in ferroptosis regulation: the new weapon for the old guardian. Cell Death Differ. 2022;29(5):895–910. doi:10.1038/s41418-022-00943-y. 35087226 PMC9091200

[28] Tan W, Dai F, Yang D, et al. MiR-93-5p promotes granulosa cell apoptosis and ferroptosis by the NF-kB signaling pathway in polycystic ovary syndrome. Front Immunol. 2022;13:967151. https://www.frontiersin.org/articles/10.3389/fimmu.2022.967151. doi:10.3389/fimmu.2022.967151. 36341347 PMC9626535

[29] Guilliams M, Scott CL. Does niche competition determine the origin of tissue-resident macrophages? Nat Rev Immunol. 2017;17(7):451–460. doi:10.1038/nri.2017.42. 28461703

[30] Berry MR, Mathews RJ, Ferdinand JR, et al. Renal sodium gradient orchestrates a dynamic antibacterial defense zone. Cell. 2017;170(5):860–874.e19. doi:10.1016/j.cell.2017.07.022. 28803730

[31] Ferenbach DA, Sheldrake TA, Dhaliwal K, et al. Macrophage/monocyte depletion by clodronate, but not diphtheria toxin, improves renal ischemia/reperfusion injury in mice. Kidney Int. 2012;82(8):928–933. doi:10.1038/ki.2012.207. 22673886

[32] Huen SC, Huynh L, Marlier A, et al. GM-CSF promotes macrophage alternative activation after renal ischemia/reperfusion injury. J Am Soc Nephrol. 2015;26(6):1334–1345. doi:10.1681/ASN.2014060612. 25388222 PMC4446881

[33] Yao W, Chen Y, Li Z, et al. Single cell RNA sequencing identifies a unique inflammatory macrophage subset as a druggable target for alleviating acute kidney injury. Adv Sci. 2022;9(12):e2103675–e2103694. doi:10.1002/advs.202103675. PMC9036000 35112806

[34] Lee DH, Park JS, Lee YS, et al. SQSTM1/p62 activates NFE2L2/NRF2 via ULK1-mediated autophagic KEAP1 degradation and protects mouse liver from lipotoxicity. Autophagy. 2020;16(11):1949–1973. doi:10.1080/15548627.2020.1712108. 31913745 PMC7595589

[35] Wang K, Long B, Zhou LY, et al. CARL lncRNA inhibits anoxia-induced mitochondrial fission and apoptosis in cardiomyocytes by impairing miR-539-dependent PHB2 downregulation. Nat Commun. 2014;5:3596. doi:10.1038/ncomms4596. 24710105

[36] Russo I, Penna C, Musso T, et al. Platelets, diabetes and myocardial ischemia/reperfusion injury. Cardiovasc Diabetol. 2017;16(1):71–81. doi:10.1186/s12933-017-0550-6. 28569217 PMC5452354

[37] Morsy MD, Mostafa OA, Hassan WN. A potential protective effect of -tocopherol on vascular complication in spinal cord reperfusion injury in rats. J Biomed Sci. 2010;17(1):55. doi:10.1186/1423-0127-17-55. 20609232 PMC2909177

